# Diversity and “Successful Aging”: Exploring Intersectional and Existential Dimensions to Aging Well

**DOI:** 10.1093/geront/gnaf008

**Published:** 2025-01-23

**Authors:** Susan Pickard, Victoria Cluley, Surinder Sharma, Upanish Oli, Gifty Thomas-Ekweh, Jay Banerjee, Chris Williams, Simon Conroy, Bram Vanhoutte, Alex Labeit

**Affiliations:** Centre for Ageing and the Life Course, University of Liverpool, Liverpool, Merseyside, UK; School of Sociology and Social Policy, University of Nottingham, Nottinghamshire, UK; Centre for Ageing and the Life Course, University of Liverpool, Liverpool, Merseyside, UK; Centre for Ageing and the Life Course, University of Liverpool, Liverpool, Merseyside, UK; Centre for Ageing and the Life Course, University of Liverpool, Liverpool, Merseyside, UK; University Hospitals of Leicester and University of Leicester, Leicestershire, UK; College of Life Sciences, University of Leicester, Centre for Medicine, Leicester, UK; MRC Unit for Lifelong Health and Ageing, University College London, London, UK; École de Santé Publique, Université Libre de Bruxelles, Brussels, Belgium; MRC Unit for Lifelong Health and Ageing, University College London, London, UK

**Keywords:** Existential phenomenology, Intersectionality, Minority ethnicity, Photovoice

## Abstract

**Background and Objectives:**

This article aims to add to the literature on successful aging in minoritized ethnic groups. Concurring with the critiques of “successful aging” for focusing on values and abilities more attainable by White middle-class older people, it explores alternative discourses according to which older people from minoritized groups consider themselves to be “aging well.”

**Research Design and Methods:**

The article draws on original empirical material derived from a longitudinal research project focused on 5 minority ethnic groups living in a city (and surrounding areas) of the UK known for its diverse population. It draws on photovoice methods conducted with the participants and analyzes the material through (i) a focus on intersectionality as a framing device that is dynamic over time and (ii) phenomenological approaches to old age as a distinct life stage existentially.

**Results:**

Although intersectionality is framed in the literature almost exclusively in terms of disadvantage, older participants made use of assets as well as deficits (which they often turned into assets) to construct a sense of meaning and purpose that enabled them to age well and flourish in often challenging circumstances.

**Discussion and Implications:**

The images and narratives presented here challenge the normative depictions of a good or successful old age constructed from the perspective of White and middle-class older adults. They add a diverse range of alternative depictions of aging well, which will be of help for clinicians and others in supporting diverse older people to flourish in conditions of health as well as frailty.

There has been little research in the field of “successful aging” regarding the experience of minority ethnic groups, including migrants, in the global north. This lack of research is situated within a global health inequalities gap, whereby it is known that older people of minority ethnicities age less successfully than their ethnic majority counterparts (Evandrou et al., 2016; Stopforth et al., 2023). In countries with aging populations, ill health strikes earlier and more severely in older people of minoritized ethnicity (Watkinson et al., 2021; Wohland et al., 2015), who also experience disadvantages, including racism that impacts on their health throughout the life course (Stopforth et al., 2022). Research does suggest a “healthy migrant effect” in some but not all populations and importantly also suggests that advantages in both health and mortality decrease with time and age (see Brabete, 2017 for an excellent summary). Understanding how people of different ethnicities experience aging, moreover, is essential to the progression of any conceptualization of “success” in older age.

However, as we will show in this article, successful aging is not a good framework for understanding what we might call “aging well,” particularly in the context of minority older people. Not only was the concept of successful aging framed to meet the criteria of White western and middle-class aging but its model, which is rather one of exceptional aging, does not consider how flourishing can take place including in conditions of frailty or life-limiting conditions. Through this framework, the health of minority older people is very much depicted in terms of deficit and failure.

In this article, we relate initial findings from an ongoing research project that explores the experience of aging, particularly aging into deeper old age (associated with frailty and the fourth age), among minority ethnic older people from a number of diverse communities living in the UK. All but one are first-generation migrants as large-scale migration to the UK from former colonies began in earnest only after World War II. However, most older people in this study have been in the UK since their youth, meaning they have been here upwards of half a century. In order to shift the focus from successful aging to the more inclusive concept of aging well, we use a lens that incorporates intersectionality, combined with an existential focus, as a multistranded theoretical framework to approach the lived experience of aging. In the first sections, we will provide an overview of the key concepts upon which we draw, beginning with the successful aging literature and, particularly, its critique.

## Successful Aging

First conceptualized by Rowe and Kahn (1987, 1997, 1998), “successful aging” differentiates usual aging from successful aging, whereby successful aging (really, then, *exceptional* aging) includes the absence of disease, the maintenance of physical and cognitive function, and productivity and psychosocial engagement. Successful aging as a concept has been widely influential (e.g., Pruchno, 2015) as well as widely critiqued (Bülow & Söderqvist, 2014). Broad criticisms in the literature include: (a) its limited construction of aging well defined within the parameters of “success,” which disregards many other elements of a valuable and good old age; and (b) relatedly, the lack of attention to the lived experiences and aspirations of older people themselves.

Despite Rowe and Kahn’s (1987) assertion that successful aging is something that can be achieved with individual effort and appropriate lifestyle choices, cumulative structural disadvantages mitigate against this for many older people (Katz & Calasanti, 2015). Moreover, if there is a focus on prevention or cure of both “usual” aging and f(r)ailed aging, then an important opportunity to support older people’s well-being, growth, and flourishing in conditions of chronic illness, life-limiting conditions, and frailty is potentially missed. Whilst the concept of successful aging encapsulates a static approach to health, in old age it seems more appropriate to consider a more dynamic condition, “based on the resilience or capacity to cope and maintain and restore one’s integrity, equilibrium, and sense of wellbeing” (Huber et al., 2011, p. 2). At the same time, western societies in particular have undergone a rapid change through migration leading to diversity and super-diversity, meaning that older people as a class are more heterogeneous than ever before, which in turn should be reflected in our views of well-being and aging well in old age.

Critiques of successful aging, including its appropriateness to minority ethnic older people, fall into two camps: either suggesting modification of the concept to accommodate a broader range of components or else discarding it altogether (Martinson & Berridge, 2015). Suggested additional components include group-specific elements such as success of adult children and a positive attitude (for South Korean older people in the United States), but these are noted to vary widely between and within cultures. The alternative frameworks include “balanced aging,” “harmonious aging,” and “resilient aging” (see Martinson and Berridge for a more detailed summary). Here, we suggest “aging well” as a broad umbrella concept to capture what diverse older people themselves define in such terms.

We next discuss what we mean by the concepts of “intersectionality” and “existential” aspects in more depth.

## Intersectionality and Standpoint

First developed by Kimberlé Crenshaw (1991) to critique the exclusion of Black female experience from second-wave feminism, intersectionality has now been applied to further the understanding of a wide range of marginalized experience. Intersectionality recognizes the role of relational characteristics, situated in systems of power that serve to oppress some and advantage others. Social identities such as age, gender, disability, social class, religion, and sexuality tend to be addressed; however, to date in intersectionality studies, age has been comparatively neglected (e.g., Weßel & Schweda, 2022). Importantly, an intersectional gaze captures the experience of difference/identity as well as inequality. As Weßel and Schweda note: “it concerns the critical engagement with social categories such as gender, class, race, and power structures that *all* people face” (2022, p. 23; our emphasis).

Although intersectionality is deployed in a variety of ways, here we use it as a framing device (Anthias, 2012), situating the individual simultaneously in their biographical context and the unique and diverse factors that structure it. We see these elements as intermeshed, fluid, and relational, rather than additive or static, working in combination to constitute unique positions (Cho et al., 2013; Collins, 1990). Although elements such as gender, race, and age are meaningful in and of themselves, they are not experienced, nor can they truly be understood, without analyzing their interaction with each other and with other factors in a particular social space and context. So for older people, depending on location and context, this can mean that some elements have more salience than others, for example, race over class and vice versa. Moreover, intersectional factors are also dynamic across the life course and as they change so they transform the relationship between the individual elements and the whole social position (Collins, 2019).

This approach is sufficiently sensitive to allow for both subjective experience and to trace the “dialogic” interplay (Anthias, 2012) between different structural elements, for example, class with race, or gender with race, or both with age across a life course. Relatedly, although the number of intersections in the literature on intersectionality has been almost invariably used to signal greater disadvantage and inequality in a straightforward cumulative equation, we reject this mechanistic approach and identify the possibility for agency through generating new and transforming older cultural capital (Erel, 2010). This kind of adaptability noted here may also help minoritized individuals, including migrants, build cultural capital over the life course including in old age.

Thus, in this article, we adopt a qualitative, interpretive methodology that links intersectionality with standpoint theory, which broadly makes the “claim, in somewhat different ways, that it is vital to account for the social positioning of the social agent” (Yuval-Davis, 2015, p. 315). Following the approach of Nancy Hartsock (1997), Dorothy Smith (1990), Patricia Hill Collins (1990), and Yuval-Davis (2015), among others, we suggest that although intersectional positioning shapes standpoint it does not determine it. Yuval-Davis observes: “Experiences, social practices, social values and the ways in which perception and knowledge production are socially organized have been seen as mediating and facilitating the transition and transformation of situatedness into knowledge” (2015, p. 316). We therefore need also to know something of an older person’s individual biography and their perception of what is meaningful, valuable, and counts as aging well to them, beyond the structural data. At the same time, there is no privileged access to the truth inherent in any one standpoint, but rather greater understanding (in this case of aging well) is to be found through a dialogical relationship among people of different positioning (Collins, 1990).

There is also a unique existential dimension to old age that is often overlooked; we turn to this next.

## From Successful Aging to Aging Well: Existential Issues

Age is often overlooked in the literature on intersectionality and this is significant not only because age leads to added (dis)advantages and inequalities but also because it is also a social location that can involve a substantive existential shift. As Eilenberger et al. (2019) observe, from a phenomenological perspective, for older people there is “an existential difference that relates to their different modes of being in the world” (2019, p. 32). Principles of phenomenology here, developed most prominently through the work of Husserl, Heidegger, and Merleau-Ponty, relate to the structures of embodied experience and include: rejection of the body/self-split germane to Cartesianism (and biomedicine) and a focus on the first-person perspective (combined with the bracketing-out of assumptions and norms possessed by the (youthful) observer). Together, these configure the experience of an embodied “intentionality” towards the world (contrasting with physical and other abilities measurable in the clinic) in which actions and objects are meaningful in terms of the goals and projects the individual may have. These factors associate the perception of “I can” with the experience of health/well-being (consonant with the idea of health suggested by Huber et al., 2011, earlier). The “I can” is structured within a “horizon” in which time and space are not experienced as objective, like clock time, but relate to lived experience and the goals and values that underpin them (see Leder, 1990; Merleau-Ponty, 1962; Toombs, 1991; and for a fuller discussion of how this relates to old age in particular, see Pickard, 2018). Thus, to understand aging well, it is necessary to understand the values and goals that have configured a life course as well as their continuity and/or change in old age.

A focus on existential issues means that we can conceptualize modes of “aging well” as alternatives to the depiction offered by “successful aging.” In a way that is particularly relevant to minority ethnicities, Drew Leder, for instance, suggests a number of cross-cultural archetypes of aging that can inspire a “cultural re-visioning” toward positive aging outside of western neoliberal discourses. He identifies a number of such positive archetypes including what he names the “contemplative,” the “contributor,” the “compassionate companion,” and the “creative.” The contemplative is a counter to the western emphasis on activity and busyness (and the focus on productivity and engagement as seen by “successful aging”). Here, Leder emphasizes the Hindu tradition where later life is the time for focusing on spirituality or more generally is a “time to simplify and refocus our life” (2018, p. 227). The contributor is focused on activity but of a kind that contrasts with that emphasized in successful aging as it includes “mentor, caregiver, and wisdom-keeper” (p. 228). The compassionate companion’s own experience of vulnerability increases sensitivity to the needs of others, and the creative suggests an individual able to reinvent themselves, taking on new roles and challenges in later life.

Bringing together the three strands of intersectionality, standpoint, and existential approaches means that we can capture subjective and agentic aspects of aging well, identify their structuring in power relations and explore both the continuities and unique aspects of the latter that prevail in old age.

## Research Design, Aims, and Methodology

This article draws on empirical findings from the first stage of a longitudinal project exploring the experience of aging among older people living in a large multiethnic city (chosen for its known diversity and large migrant population in the UK). Eligibility criteria for the study itself included older people (age 50 and older) with capacity to consent, from one of six ethnic groups (Bangladeshi, Pakistani, Black Caribbean, Black African, Indian, and White). The lower age threshold was intended to ensure inclusion of certain ethnicities who may experience aging and frailty at earlier ages than others (Nazroo, 2022, p. 2). However, in practice, all participants were 60 years and older, indeed being aged 63–86. In this article, we draw only on the data concerning the minority ethnic participants. Participants were recruited using a mixture of purposive and convenience sampling through community centers, lunch clubs, and faith groups, among others. The fieldwork is led by X with the assistance of two research assistants (RAs) recruited from the local minority communities (Y and Z), where Y provided translation for those of South Asian heritage unable to speak English (supplemented by community translators in the case of the Bangladeshi community).

The first phase of our research, which we discuss in this article, involves photovoice.

### Photovoice

Photovoice is an inclusive and accessible research method that allows participants to both show and tell their experiences (Cluley, 2021). Initial data collection is devolved to the participant, through the request to take photographs of a given subject/experience/event. Participants are encouraged to share images of what they choose to capture about their worlds, rather than be directed by researchers. For this reason, photovoice is particularly useful in drawing out the unique standpoint involved in both intersectional identities and existential meaning.

**Figure 1.**
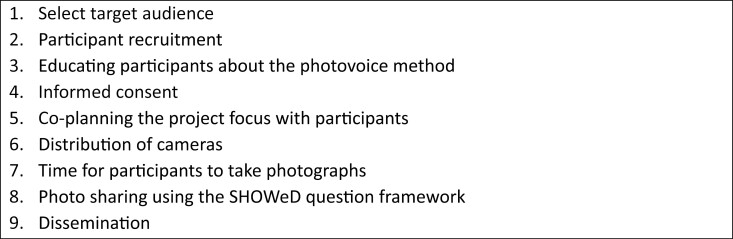
Nine steps of photovoice.

Photovoice is a flexible research method but typically involves a combination of nine steps (Wang & Burris, 1997; see Figure 1). A typical photovoice study involves participants taking photographs of a negotiated subject, sharing the photographs with the researcher, and using the photographs to guide a conversation and facilitate the discussion of ideas to promote change. Importantly, the images in a photovoice study are not supplements to a semistructured interview, rather they are a means to the elicitation and understanding of experience. Owing to the significance of this visual/verbal relationship, photovoice is often framed in terms of an inclusive research method and consequently has been used in a range of studies to capture the experiences of marginalized groups including older people generally (Mysyuk & Huisman, 2020) and older immigrants (Brotman et al., 2020). Limitations include difficulties in taking photographs due to problems with mobility, vision, and/or functional ability, which we sought to mitigate by offering help with taking photographs whilst family members and friends also helped where needed.

**Figure 2.**
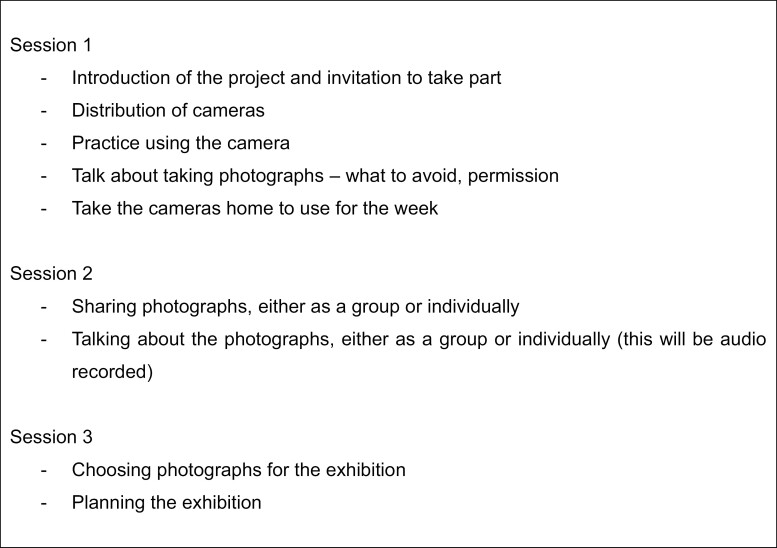
Photovoice sessions.

Seventy-three participants across six different ethnicities (Black African, Black Caribbean, Indian, Pakistani, Bangladeshi, and White British) were recruited to the photovoice study (see Table 1, for sample specifics) from local community groups specifically for older people. X approached specific groups in the locality to seek permission to recruit service users as research participants. Once permission was granted, X, Y, and Z attended group sessions to meet service users and introduce the study. They attended several times before recruitment to get to know service users and build trust. After several visits, all service users attending the group were invited to take part in the study. Participants were given a participant information sheet to take home. X, Y, and Z then attended the group session to answer questions and gain written consent. The total number of participants invited to take part was not counted, however, 73 were recruited and 68 took part and were present at all sessions. Reasons for not taking part included lack of time and/or interest, and forthcoming holidays. The photovoice work took place over 6 months (between May and November 2023). Seven groups of participants attended photovoice sessions over two to three arranged sessions. These sessions followed the same format and were designed to encourage a supportive environment and foster a relationship of trust and respect (see Figure 2 for a breakdown of session content). Participants were given an open brief to photograph anything and everything that illuminated their experience of aging over the course of a week. Some participants expressed concern that their lives would be “too boring” but were reassured that nothing would be considered too boring and that everyday experiences were the focus. A simple, point-and-shoot, digital camera was given to all participants, and time in session one was allowed for participants to practice using them. Only one participant struggled to use the camera and did not produce any photographs for this reason. Additionally, each participant was asked to fill in, with the help of our community RAs, Y and Z, a frailty index questionnaire designed to determine levels of frailty.

**Table 1.** Sample Specifics.

Ethnicity	Total number of participants	Gender
Black Caribbean	10	Female (8) Male (2)
Black African	6	Female (3) Male (3)
Indian	14	Female (10) Male (4)
Bangladeshi	15	Female (7) Male (8)
Pakistani	13	Female (11) Male (3)
White British	10	Female (7) Male (3)

**Figure 3.**
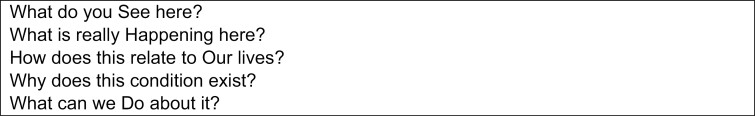
SHOWeD questions.

The photo-sharing sessions were facilitated by X, the qualitative lead for the project, with Y and Z. A flexible topic guide was used to facilitate talk about the images informed by the SHOWeD framework of questions (see Figure 3) together with a number of additional questions that asked participants what they felt about the photographs and how this related to their health or other aspects of their lives, either directly or indirectly. These questions facilitated further probing questions based on the participant’s answers. In this way, conversations about the photographs covered a wide range of topics such as health, aging well, migration, and support networks. The photovoice sessions were audio recorded and transcribed. Talk about the photographs that took place either in groups or individually, depending on individual preference and circumstance.

### Analysis

Although photovoice provides an accessible method that was well suited to this study, the photovoice method does not provide a particular framework for analysis. Indeed, frameworks for the analysis of visual data are few and far between (Cluley et al., 2021; see Drew & Guillemin, 2014; Liebenberg et al., 2012 for examples).

**Figure 4.**
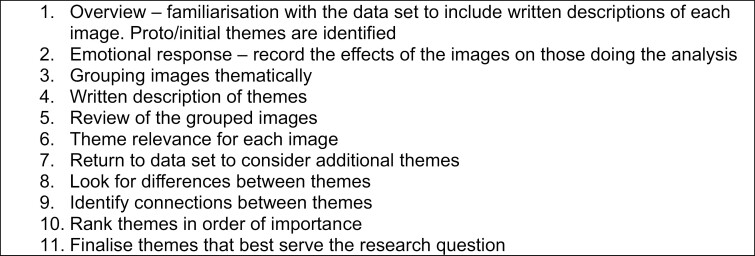
Polytextual analysis.

**Figure 5.**
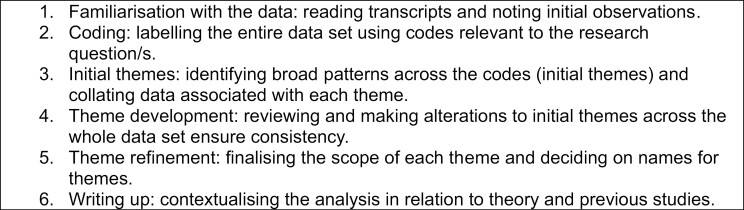
The six phases of thematic analysis.

Following Whitfield et al.’s (2023) lead, X and W used a combination of polytextual (Gleeson, 2012) and reflexive thematic analysis (Braun & Clarke, 2023), taking ideas from both to analyze the initial findings of our photovoice data (both images and talk). Polytextual analysis provides an iterative framework to facilitate the rigorous analysis of visual data. Described by Gleeson (2012) as a recipe, polytextual analysis involves 11 steps (see Figure 4 for a full list). Similarly, reflexive thematic analysis (Braun et al., 2023) provides a rigorous framework for analyzing textual data (see Figure 5 for further details). This combination allows the content and context of both forms of “data” (the images and the transcripts) to be considered together. In this article, we focus on the participants’ perceptions and experience of aging and relate this to the idea of “aging well.”

Our approach took three stages. In the first, we modified step one of the polytextual analysis recipe to better match the steps of reflexive thematic analysis. Rather than identifying protothemes at this stage, we coded the photographs based on their content and the context we gained from the transcripts. The codes included recurring elements such as pets, natural environment, family, and food but also more contextual information such as age, gender, and racialized experiences. Images were often assigned multiple codes. We also avoided assigning themes according to a hierarchical order (step 10), instead allowing the importance to be identified by individuals. X and W coded the images and transcripts together and engaged in a number of iterative analysis sessions to develop protothemes. Owing to the combined textual and visual nature of photovoice, the significance of the visual and the spoken is both equal and constituent. Thus, we did not prioritize findings from one or the other but sought to combine the findings to build a holistic understanding of aging across the participant groups.

In the second stage, we used an abductive approach to coding the photovoice transcripts (Thompson, 2022), whereby we started with preset codes based on current literature on aging well, intersectionality, and phenomenology and added to this as further codes were identified in the transcripts. The codes included health experiences, aging, approach to aging, changes in relation to aging, hobbies, aides and adaptations, planning for the future, mobility, support networks, family, personal values, mindset, community groups, and animals and nature. X and W coded half of the transcripts each and then met to share codes and discuss discrepancies, overlaps, and potential themes. X then checked all transcripts to ensure continuity.

In the last stage, several key themes were identified from both sources of data through an iterative and collective approach whereby X and W first grouped codes that were similar and then discussed the salient points, similarities, and differences between each group of codes. Themes were identified through this process of moving back and forth between the code groups to identify key messages in relation to the study aims. These cross-cutting themes include the importance of religious belief and practice, meaning/purpose, belonging, connection with other generations and generativity, and feeling heard.

**Figure 6.**
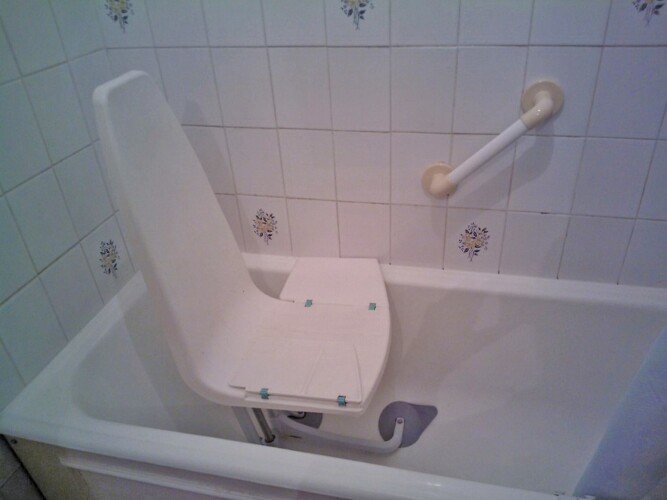
These aids and adaptations “were all done for my husband but while they were doing it, I knew that I would probably need all these things [in the future].”

**Figure 7.**
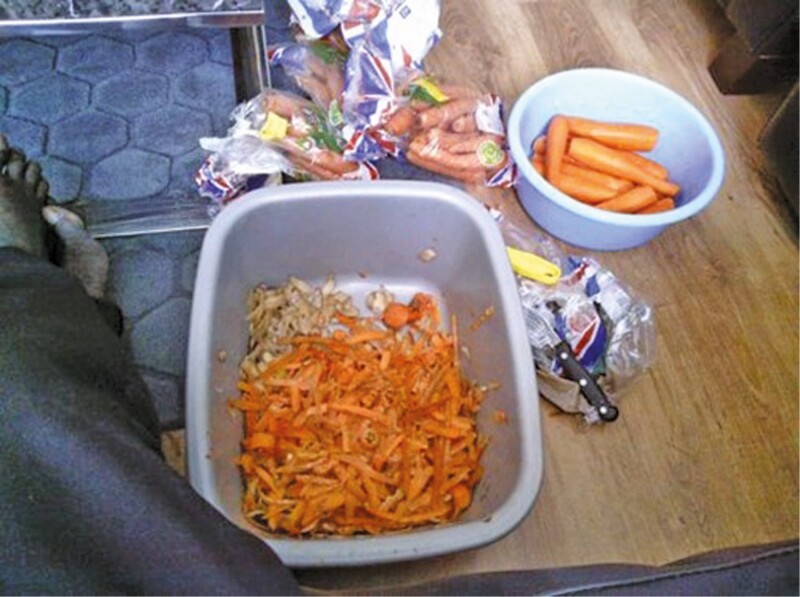
“[T]his peeler… resembles what we call a microtome in the medical field.”

**Figure 8.**
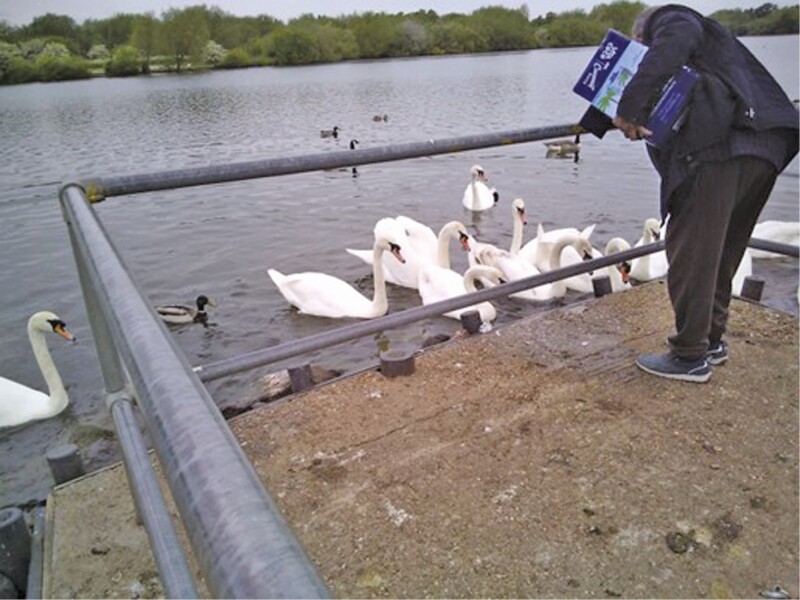
“I love them—the thing is they are innocent…”

**Figure 9.**
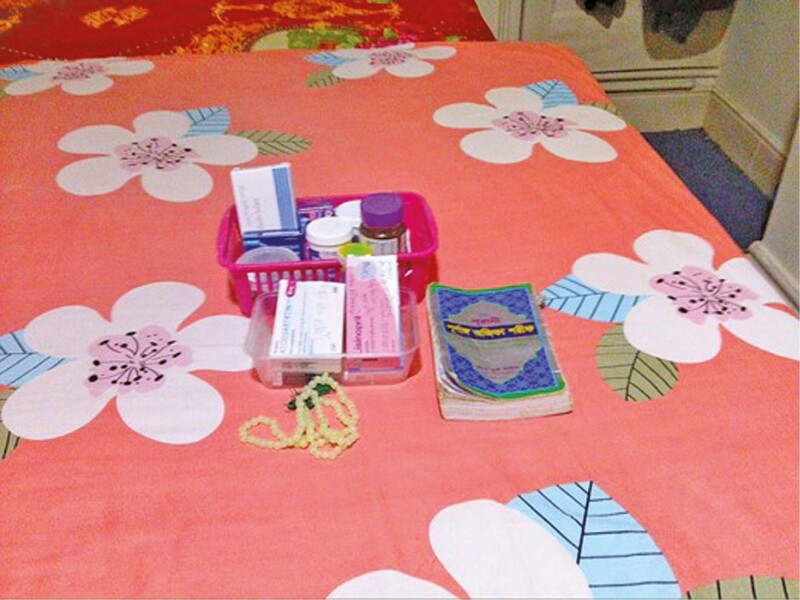
“[P]raying brings peace to my heart.”

Although these themes were present across each minoritized group, in practice each element was interpreted very differently by participants. Thus, in order to focus on this heterogeneity within a range of minority ethnic communities, we present four case studies via a mixture of photograph and textual data. These were selected in order to include a mix of intersectional details including ethnicity, gender, social class, religion, marital status, and so on, as well as health status. Because of their age and the history of migration from former colonies to the UK, as noted, all were first-generation migrants, but three out of the four had been living in this country between 40 and 60 years and had raised their families in the UK. The photographs that accompany these texts (Figures 6–9) were chosen by the participants themselves as the most meaningful of all the photographs they had taken.

Our four case studies illustrate how these cross-cutting themes combine uniquely to enable different experiences of aging well, including in conditions of chronic illness and disability and in turn can be brought into relationship with each other in the dialogic pattern highlighted earlier. Each case study is presented as a vignette to provide a descriptive account of the individual participant’s life course and the intersectional and existential factors of importance to it. Each vignette links to the themes identified in our analysis. Finally, as we discuss at the end of the article, the findings can be related to the literature on successful aging and illustrate the need to broaden and diversify our understanding of health and well-being in old age.

## Findings: Diverse Ways of Aging Well

### Aging Well for Ines: Helping Self and Others and Combating Injustice

Ines is an 86-year-old woman who migrated to the UK from Jamaica in the 1950s. Ines grew up in a middle-class family and was privately educated. Having trained and worked as a nurse in Jamaica, Ines responded to the call for healthcare workers from Caribbean islands to work in the UK, where she worked in various nursing and healthcare management roles until retirement. Ines met her husband in the UK, who had also migrated from a Caribbean Island and was also from a middle-class family. Having qualified professionally in Jamaica, he was obliged to practice his professional work there, owing to structural barriers to recognizing his accreditation in the UK, meaning that he commuted between both countries for many years. They had three children, one of whom was stillborn, and remained together until he died 10 years ago. Ines lives alone in the house in which she raised her children and later cared for her husband. She has multiple chronic illnesses that cause her continuous pain, and her mobility is deteriorating, so she now uses furniture to move around the house and keeps a walking stick in each room. Her frailty score positioned her as mildly frail.

Ines excelled in her career. She served as chairperson of the local branch of her union, campaigning for nurses rights, and for the professionalization of nursing. Ines continues the values associated with her identity as a professional into retirement. She also cares for and guides both family members and others. Whilst Ines’ children live in other countries now and both have successful careers, grandchildren would stay with Ines for the entire summer break. When Ines’ husband was diagnosed with dementia, Ines cared for him at home. In addition, Ines is a lifelong Baptist and is heavily involved in her local Baptist church community, volunteering in various pastoral roles.

Ines shared five photographs, all of which showed either home accessibility adaptations or home security aids such as a falls alarm.

The adaptations, including that shown in Figure 6 earlier, had been put in place to support her husband following his diagnosis of dementia and subsequent deteriorating mobility. Although Ines did not need these aids at the time of her husband’s illness, she nevertheless retained them in anticipation of future needs. She is now finding them helpful and indeed they constitute a key element of her own self-care.

Education of self and others has been a lifelong theme for Ines. She retired in a senior management role and talked about the grit and determination that this involved, linking this to her ethnicity and the racism that she faced early in her career. For example, as a Black nurse in the NHS when she first moved to the UK, people would ask, “did I wear a grass skirt and did I live in a tree?” She discussed being awarded lower marks in her exams than White nurses, although she knew she was more capable and worked harder.

Ines used the racism and sexism she witnessed and experienced as motivation to improve things for others. This ability to forge ahead despite obstacles builds partly on her cultural capital as a middle-class woman in her country of origin and also on dispositions of courage and strength, which she displayed in choosing to migrate as a younger woman. In one vivid example, Ines relates how she was able to transform an experience both of racism and of gendered inequality into an opportunity to redress institutional shortcomings for the benefit of all. Following her own painful experience of having a stillbirth and being treated carelessly, when she occupied a managerial role, Ines organized a support service for mothers of stillborn children including offering a side room in the maternity ward of the hospital that could be used for such women, which is still in use today.

This theme has transformed its focus in old age from campaigning and social justice work within the professional organization to helping her community, particularly her church community. She has observed how many of her Black peers, lacking her own professional experience and cultural capital, lack information about healthcare processes.

I take my professional life to church as well, I give advice to members, information. They all know I’m a nurse, so we discuss, we don’t talk about Jesus all the time, we discuss health and … I give them information.

With her main social interaction today being in her church and family, Ines says she experiences less racism, a factor that further facilitates her sense of well-being and flourishing at this point in her life. Yet she continues to witness and challenge the effects of racism and exclusion as it affects others through “sharing and giving information … as our Black community we do not get enough information about what’s out there … to help as an old person.” This includes sharing information about self-help in terms of vitamins and health regimes, also about dementia, which she had learned through caring for her husband, using her own vulnerability to empathize with others. She takes old age as a particular challenge, sharing that she dressed each day “as if she is going to work—because old age *is* work.” In turn, she derives meaning, value, a sense of being heard, and ongoing self-esteem from her role.

### Aging Well for Charles: Adaptability and Lifelong Learning and Teaching

Charles is a 70-year-old retired doctor and medical educator originally from West Africa. He has lifelong hypertension and a recent onset of type 2 diabetes and was identified as mildly frail on our scale. He fled one African state as a refugee at a time of political instability, completing the medical education he had started in this new country where he went on to qualify as a consultant. In his thirties, he moved to the UK to teach at a medical school. Leaving behind upper middle-class comfort, which included three servants at one point, he settled in a modest and predominantly White area of the city in which he has lived ever since. Charles is a lifelong Christian and attends weekly Pentecostal church meetings. He describes his life very much in terms of an orientation to the future including generativity. He chooses to do the housework so his (now adult) children can focus on their work and on “their future” (they are both young professional scientists), and one of the photographs he chooses is that of him peeling vegetables, sitting in the living room with a plate on his knees. This is an interesting picture for several reasons (Figure 7). The peeler fascinates him, he says, through its similarity to a specialist surgical instrument. Second, it gives him the opportunity to discuss how he adapted the traditional gender roles expected in West African society. He reflects:

In Africa, in my tribe in particular, if a man is seen in the kitchen, it’s a taboo, you’re not supposed to go into the kitchen, you are not supposed to start cooking because it is disrespectful to do the work which the women are supposed to do.

Hence, Charles stays out of the kitchen but does the prepping for his wife and daughter and indeed, takes great pride in his contribution especially as he is able to link it to his interest in technologies and instruments, emphasizing the way the lifelong theme of adaptability continues to play out in old age. Similarly, since retirement he has taken up the role of ironing but emphasizes how the steam iron he uses is a very interesting technical gadget whose design he discusses at length with the interviewers.

In his retirement, he continues to focus on development of self and others. He watches documentaries and history programs and feels he is learning many things he lacked time for during his working days. He continues his long-term interest in mentoring and helping develop the skills of doctors back in his home country. The project currently absorbing him involves developing an innovative computer program to help orient medical students toward clinical knowledge. This is sustaining for him and makes his later life very meaningful. Without this project, he acknowledges, his retirement “would have been very miserable.”

Yet, he admits he has no support from the university where he used to work:

Unfortunately… when you’re a Black person—I must say this—when you’re a Black person nobody seems to trust your knowledge.

Although like Ines, he highlights the importance of his Christian faith. Charles’ focus is more on the community aspect, and he makes his important contribution through applying his technical skills, including setting up speakers for discos and lighting for shows.

### Aging Well for Amit: Connecting With Nature and Living by Hindu Principles

Amit is a 68-year-old Hindu man who lives with his wife of nearly 50 years and one of their adult sons. Amit has a number of chronic health conditions that impact on what he can and cannot do, and his wife now acts as his carer. Amit has severe arthritis in his knees, ankles, hips, and back, which limits his mobility considerably. Amit also has type 2 diabetes, high blood pressure, chronic diverticulitis, and what he described as “some emphysema.” He was positioned on our scale as severely frail. The embarrassment Amit feels about his diverticulitis severely reduces the activities he will engage in and time periods during which he will leave the house.

Amit moved to the UK from Kenya with his family over 50 years previously, when he was a teenager when his small-business-owning father was no longer permitted to work there by the new government. During his working life he was a small-business owner in the clothing industry. X met Amit at a social group for older people that ran on a weekly basis in a community center in a local area predominantly populated by Indian migrants from East Africa. Amit had been attending the group for some years, and he and his wife have recently signed up for Bollywood singing classes. Through photovoice, he describes a few things of key importance to him today: his faith (he is a Brahmin Hindu) and allied to that his vegetarianism, his Indian heritage more broadly and his connection with animals and nature. Indeed, many of Amit’s photographs showed his dog Tiger. Explaining one photograph, Amit said, “She [Tiger] makes sure that I’m fine, you know, even during the day if I’m sitting and I nodded off or my breathing has got shallow, she’ll come and nudge me.”

Amit’s Hinduism has become even more important to him in this life stage, and a photograph of religious objects instigated a discussion about this. Amit said

The thing is, if you believe in faith, right, it’s like a law abiding citizen, if you don’t have faith you’ve got no guidance, no rules, no regulations, nothing. Then it becomes like chaos.

Having a strong faith that the course of his life was predetermined from birth helped Amit to make sense of the aging process and limitations and ill health he had faced so far.

Amit’s religious principles also shape his attitude toward animals. As well as his close bond with his dog, this photograph shows Amit at his local park feeding the swans and ducks. Amit regularly drives to the park and walks the short distance to the lake, using the rails on the jetty first to help him walk, then for support when standing and feeding the ducks. When talking about this, Amit also discussed the importance he attributed to making regular trips to India, despite the challenges this presented for someone in poor health:

I love going to and feeding them. When I’m in India I stay a month and we’ve got a shop not far from the hotel, so when I go, I always buy coconut cream for them [the ducks]. I love them.

### Aging Well for Bakhul: An Old Age Structured by Prayer and Family Life

Bakhul is a 63-year-old Bangladeshi woman who migrated to the UK from Italy 6 years ago. Bakhul had lived in Italy for just over a decade and moved from Bangladesh to join her husband, who had migrated to find work. Neither Bakhul nor her husband have professional skills and struggled to find work in Bangladesh. In Italy, Bakhul’s husband worked in a pizza restaurant and when Bakhul joined him with their children she worked as a housewife. When their children finished school in Italy, they moved to the UK and Bakhul and her husband moved to join them utilizing the EU settlement scheme. Bakhul’s husband died 2 years ago, and she now lives with one of their sons and his wife. Bakhul has numerous health conditions, limited mobility, and relies on others to take her out and was classified as severely frail. In addition to this, Bakhul speaks very limited English and relies on her children for translation. Consequently, Bakhul spends most of her time at home, sitting in the living room, going out mainly for health-related appointments, and once a week to a lunch club.

X met Bakhul at the lunch club she attends and spoke to her with the help of a translator. Bakhul’s photographs showed many images of her living room, where she spends most time, religious artifacts, and medication. Image four shows a composition that Bakhul had put together to show the important things in her life. When talking about this image, Bakhul talked about how her time in old age is now organized around her commitment to prayer and her need for timed medication. This led to discussion about Bakhul’s living arrangements and her expectations for aging well.

For Bakhul, aging well is a life structured by faith and family relationships especially based on her cultural expectations of multigenerational living. Bakhul prays 8–9 times a day using the Koran, prayer books, and prayer beads shown in Figure 9. Bakhul talked about the judgment that would be made about her life upon death and the role prayer played in this. For Bakhul, “good deeds” included living morally, giving to charity, helping out in the community, praying, and ensuring she lives by the tenets of her faith. Bakhul said:

I can’t help out in the community anymore but I pray more to make up for that. I can pray more throughout the day because I am not so busy as I used to be.

Bakhul discussed living in a multigenerational household and deriving enormous satisfaction from having a daughter-in-law, meaning that she now cooks and cleans very little, choosing when and if she would like to do these tasks rather than feeling like she is required to. For Bakhul, who had cooked, cleaned, raised children, and looked after older relatives (as a daughter-in-law herself), attaining this position was viewed as an achievement that she had worked her entire adult life in anticipation of (our translator mentioned that this was less common today among younger generations). Similarly, although she sat in one chair in the living room all day, this did not feel like a diminishment, and indeed it feels like a very full life because she could partake in every family activity from there and also carry out her religious activities from the chair. Here, then, Bakhul’s life in old age is very much more than the sum of its parts; cultural and individual life course factors indicate positive elements where an intersectional focus, although important, might see only disadvantage and deficit.

## Discussion and Conclusion

In these diverse accounts we can view the dynamic nature of intersectional relations and unique biographical factors through the life course, with old age as a unique existential stage structured both by continuity and difference from the rest of the life course. Ines, for instance, has moved from professional leadership based on challenging experiences to leadership in her church and community. Her sunny but determined disposition, based on a commitment to social justice and care for others has not changed but it has been diverted into different channels. We can consider these case studies through Leder’s positive archetypes of aging. Whilst Ines’ experience of aging fits very much with the contributor archetype, there are also elements of the compassionate companion here too, with her experience of caring for her husband, as well as her own suffering, underpinning a shared vulnerability. By contrast, Charles’ resilience is of a different kind, his life having been characterized by adaptability, in gender, class, and race terms. His flourishing has also always consisted of generativity and legacy-building and in older age he continues to be a contributor and also to add a more “creative” aspect where his magnum opus is both conceived as his ultimate legacy and as something that makes his retirement meaningful.

For all of the older people, faith is of key importance, but for Amit and Bakhul it provides most of the meaning and structure to this life stage. Whilst Amit claims a lifelong Hinduism, his faith is the factor that has enabled continuity where few of the concerns or interest associated with his working life have been carried over into old age, meaning that he experienced the end of his working life as a sharp break. In an old age that contains the experience of chronic illness, faith underpins a switch from an active approach to life to a more contemplative one. In Bakhul’s case, contemplation is underpinned by very limited resources as well as minimal integration in broader society. But for her, faith, combined with a sense of having reached a desirable position in the family hierarchy fosters a sense of satisfaction and indeed, in her own perception, flourishing in the midst of social deprivation and ill health. Indeed, where the older two participants here, Ines and Charles, can be positioned in the “third age” and the younger two (Amit and Bakhul) broadly in the fourth (both these concepts being arguably more applicable to a White western demographic), nevertheless flourishing is a feature of all four.

Our research has implications for policy and practice. In place of the template of successful aging, which is in fact both exceptional and modeled on a western template, we offer diverse images of flourishing in later life derived from the accounts of older people from marginalized communities in the UK, including those who have been living in the country for many decades and more recent arrivals. In so doing, we stress a more heterogeneous notion of what it is to age well, what it feels and looks like, and what factors support or undermine it, where the danger currently is that hegemonic notions of successful aging make this difficult to recognize importantly in interactions with health and social care professionals. This in turn will facilitate the cultural competence of professionals working in super-diverse societies. Additionally, it can bolster a broader dialogic understanding of the rich and complex factors that comprise what it is to “age well,” which can then be supported appropriately. In this we agree with previous research that the most significant element of aging well among minoritized older people is its diversity and heterogeneity (Martinson & Berridge, 2015). Accordingly, we also agree with the authors regarding not only successful aging’s lack of appropriateness to minoritized older people but its potential harmfulness: ideal models presuppose that there is one agreed-upon set of criteria that is appropriate for old people to strive toward (2015, p. 66). At the same time as stressing the importance of age as an intersection, we also stress the uniqueness of old age phenomenologically. Following Eilenberger et al.’s (2019) call, we have aimed to bring together existential and intersectional elements in order to facilitate a richer understanding of the things that matter in the context of health, well-being, and frailty in old age.

Limitations include the small sample size from each ethnic grouping which, combined with heterogeneity within each grouping, militates against generalizability. However, illuminating the complex heterogeneity is itself useful in guarding against stereotypical or tacit assumptions. This article presents findings at an early stage in our research and through the next stage of our project involving longitudinal interviews we intend, among other things, to explore in more detail how these elements shift over time, in relation to each other and in response to changes in social and material circumstances including health. We hope also to be able to uncover further positive archetypes of aging that resonate in a multicultural and diverse setting. The multifaceted data will also enable us to further explore the relationship between frailty, well-being, and an intersectional life course perspective (Ferrer et al., 2017). This will not only democratize our vision of successful aging but will also aid clinicians, policymakers, and others in understanding the complicated interaction of biological, material, and existential elements implicated in adverse events such as falls and hospitalization as well as robustness and resilience.

## Data Availability

Fully anonymized original data collected during the project, as well as codes generated to analyze the data, will be available from January 1, 2026, following completion of the project. These are accessible through our study website at https://www.liverpool.ac.uk/sociology-social-policy-and-criminology/research/research-projects/frailty-and-ethnicity/ and in the University of Liverpool Research Data Catalogue at https://datacat.liverpool.ac.uk/ Ahead of that date, please contact the PI, Susan Pickard.

## References

[CIT0001] Anthias, F. (2012). Intersectional what? Social divisions, intersectionality and levels of analysis. Ethnicities, 13(1), 3–19. https://doi.org/10.1177/1468796812463547

[CIT0002] Brabete, A. C. (2017). Examining migrants’ health from a gender perspective. In M. P. Sánchez-López & R. M. Limiñana-Gras (Eds.), The psychology of gender and health (pp. 231–250). Academic Press. https://doi.org/10.1016/B978-0-12-803864-2.00008-0

[CIT0003] Braun, V., Clarke, V., Hayfield, N., Davey, L., & Jenkinson, E. (2023). Doing reflexive thematic analysis. In S. Bager-Charleson & A. McBeath (Eds.), Supporting research in counselling and psychotherapy: Qualitative, quantitative, and mixed methods research (pp. 19–38). Springer International Publishing. https://doi.org/10.1007/978-3-031-13942-0_2

[CIT0004] Brotman, S., Ferrer, I., & Koehn, S. (2020). Situating the life story narratives of aging immigrants within a structural context: The intersectional life course perspective as research praxis. Qualitative Research, 20(4), 465–484. https://doi.org/10.1177/1468794119880746

[CIT0005] Bülow, M. H., & Söderqvist, T. (2014). Successful ageing: A historical overview and critical analysis of a successful concept. Journal of Aging Studies, 31, 139–149. https://doi.org/10.1016/j.jaging.2014.08.009 25456631

[CIT0006] Cho, S., Crenshaw, K. W., & McCall, L. (2013). Toward a field of intersectionality studies: Theory, applications, and praxis. Signs: Journal of Women in Culture and Society, 38(4), 785–810. https://doi.org/10.1086/669608

[CIT0007] Cluley, V., Pilnick, A., & Fyson, R. (2021). Improving the inclusivity and credibility of visual research: Interpretive engagement as a route to including the voices of people with learning disabilities in analysis. Visual Studies, 36(4-5), 524–536. https://doi.org/10.1080/1472586x.2020.1807402

[CIT0009] Collins, P. H. (1990). Black feminist thought: Consciousness and the politics of empowerment. HarperCollins.

[CIT0008] Collins, P. H. (2019). Intersectionality as critical social theory. Duke University Press. https://doi.org/10.1215/9781478007098

[CIT0010] Crenshaw, K. (1991). Mapping the margins: Intersectionality, identity politics, and violence against women of colour. Stanford Law Review, 43, 1241–12. https://doi.org/10.2307/1229039

[CIT0011] Drew, S., & Guillemin, M. (2014). From photographs to findings: Visual meaning-making and interpretive engagement in the analysis of participant-generated images. Visual Studies, 29(1), 54–67. https://doi.org/10.1080/1472586x.2014.862994

[CIT0012] Eilenberger, H. G., Halsema, A., & Slatman, J. (2019). Age difference in the clinical encounter: Intersectionality and phenomenology. American Journal of Bioethics, 19(2), 32–34. https://doi.org/10.1080/15265161.2018.1557280 31543015

[CIT0013] Erel, U. (2010). Migrating cultural capital: Bourdieu in migration studies. Sociology, 44(4), 642–660. https://doi.org/10.1177/0038038510369363

[CIT0014] Evandrou, M., Falkingham, J., Feng, Z., & Vlachantoni, A. (2016). Ethnic inequalities in limiting health and self-reported health in later life revisited. Journal of Epidemiology and Community Health, 70(7), 653–62. https://doi.org/10.1136/jech-2015-206074 26787199 PMC4941192

[CIT0015] Ferrer, I., Grenier, A., Brotman, S., & Koehn, S. (2017). Understanding the experiences of racialized older people through an intersectional life course perspective. Journal of Aging Studies, 41, 10–17. https://doi.org/10.1016/j.jaging.2017.02.001 28610750

[CIT0016] Gleeson, K. (2012). Polytextual thematic analysis for visual data. Visual methods in psychology: Using and interpreting images in qualitative research, In P. Reavey (Ed.), A handbook of visual methods in psychology (pp. 536–554). Routledge. https://doi.org/10.4324/9781351032063-3631

[CIT0017] Hartsock, N. C. (1997). Comment on Hekman’s “truth and method: Feminist standpoint theory revisited”: Truth or justice? Signs: Journal of Women in Culture and Society, 22(2), 367–374. https://doi.org/10.1086/495161

[CIT0018] Huber, M., Knottnerus, J. A., Green, L., Van Der Horst, H., Jadad, A. R., Kromhout, D., & Smid, H. (2011). How should we define health? British Medical Journal, 343, d4163. https://doi.org/10.1136/bmj.d4163 21791490

[CIT0019] Katz, S., & Calasanti, T. (2015). Critical perspectives on successful aging: Does it “appeal more than it illuminates?” Gerontologist, 55(1), 26–33. https://doi.org/10.1093/geront/gnu027 24747713 PMC4986584

[CIT0020] Leder, D (1990). The absent body, Chicago University Press.

[CIT0021] Leder, D. (2018). What is it to “age well?” Re-visioning later life. In K. Aho, (Ed.), Existential medicine: Essays on health and illness (pp. 223–234). Rowman and Littlefield.

[CIT0022] Liebenberg, L., Didkowsky, N., & Ungar, M. (2012). Analysing image-based data using grounded theory: The negotiating resilience project. Visual Studies, 27(1), 59–74. https://doi.org/10.1080/1472586x.2012.642958

[CIT0023] Martinson, M., & Berridge, C. (2015). Successful aging and its discontents: A systematic review of the social gerontology literature. Gerontologist, 55(1), 58–69. https://doi.org/10.1093/geront/gnu037 24814830 PMC4986586

[CIT0024] Merleau-Ponty, M. (1962). Phenomenology of perception. Routledge.

[CIT0025] Mysyuk, Y., & Huisman, M. (2020). Photovoice method with older persons: A review. Ageing and Society, 40(8), 1759–1787. https://doi.org/10.1017/s0144686x19000242

[CIT0027] Nazroo, J. (2022). Race/ethnic inequalities in health: Moving beyond confusion to focus on fundamental causes. Institute for Fiscal Studies. Available at https://ifs.org.uk/inequality/wp-content/uploads/2022/11/Race-health-inequalities-final-IFS-Deaton-Review-Inequality.pdf

[CIT0028] Pickard, S. (2018). Health, illness and frailty in old age: A phenomenological exploration. Journal of Aging Studies, 47, 24–31. https://doi.org/10.1016/j.jaging.2018.10.002 30447866

[CIT0029] Pruchno, R. (2015). Successful aging: Contentious past, productive future. Gerontologist, 55(1), 1–4. https://doi.org/10.1093/geront/gnv002 26035905 PMC4986598

[CIT0030] Rowe, J. W., & Kahn, R. L. (1987). Human ageing: Usual and successful. Science, 237, 143–149. https://doi.org/10.1126/science.3299702 3299702

[CIT0031] Rowe, J. W., & Kahn, R. L. (1997). Successful ageing. Gerontologist, 37, 433–440. https://doi.org/10.1093/geront/37.4.433 9279031

[CIT0032] Rowe, J. W., & Kahn, R. L. (1998). Successful ageing. Random House.

[CIT0033] Smith, D. (1990). The conceptual practices of power: A feminist sociology of knowledge. Northeastern University Press.

[CIT0034] Stopforth, S., Kapadia, D., Nazroo, J., & Bécares, L. (2022). The enduring effects of racism on health: Understanding direct and indirect effects over time. Social Science and Medicine—Population Health, 19, 101217. https://doi.org/10.1016/j.ssmph.2022.101217 PMC9450139 36091297

[CIT0035] Stopforth, S., Kapadia, D., Nazroo, J., & Bécares, L. (2023). Ethnic inequalities in health in later life, 1993-2017: The persistence of health disadvantage over more than two decades. Ageing and Society, 43(8), 1954–1982. https://doi.org/10.1017/S0144686X2100146X

[CIT0036] Thompson, J. (2022). A guide to abductive thematic analysis. Qualitative Report, 27(5), 1410–1421. https://doi.org/10.46743/2160-3715/2022.5340

[CIT0037] Toombs, S. K. (1991). Reflections on bodily change: The lived experience of disability. In S. K. Toombs (Ed.), Handbook of phenomenology and medicine (pp. 247–261). Springer. https://doi.org/10.1007/978-94-010-0536-4_13

[CIT0040] Wang, C., & Burris, M. A. (1997). Photovoice: Concept, methodology, and use for participatory needs assessment. Health Education & Behavior, 24(3), 369–387. https://doi.org/10.1177/109019819702400309 9158980

[CIT0038] Watkinson, R. E., Sutton, M., & Turner, A. J. (2021). Ethnic inequalities in health-related quality of life among older adults in England: Secondary analysis of a national cross-sectional survey. Lancet Public Health, 6(3), e145–e154. https://doi.org/10.1016/S2468-2667(20)30287-5 33516278

[CIT0039] Weßel, M., & Schweda, M. (2022). Recognizing the diverse faces of later life: Old age as a category of intersectional analysis in medical ethics. Journal of Medicine and Philosophy, 48(1), 21–32. https://doi.org/10.1093/jmp/jhac038 36519751

[CIT0041] Whitfield, E., Parnell Johnson, S., Higgs, P., Martin, W., Morgan-Trimmer, S., Burton, A., Poppe, M., & Cooper, C. (2023). Nature as a “lifeline”: The power of photography when exploring the experiences of older adults living with memory loss and memory concerns. Gerontologist, 63, 1672–1682. https://doi.org/10.1093/geront/gnad126 37793397 PMC10724040

[CIT0042] Wohland, P., Rees, P., Nazroo, J., & Jagger, C. (2015). Inequalities in healthy life expectancy between ethnic groups in England and Wales in 2001. Ethnicity and Health, 20(4), 341–53. https://doi.org/10.1080/13557858.2014.921892 24897306 PMC4648377

[CIT0043] Yuval-Davis, N. (2015). Situated intersectionality and social inequality. Raisons Politiques, 58(2), 91–100. https://doi.org/10.3917/rai.058.0091

